# Supplementary material for: Embryos assist morphogenesis of others through calcium and ATP signaling mechanisms in collective teratogen resistance
Source: Nat Commun. 2024 Jan 17;15:535. doi: 10.1038/s41467-023-44522-2 (PMC10794468; doi:10.1038/s41467-023-44522-2)
Supplement: Supplementary file 1 — Supplementary Information [file 41467_2023_44522_MOESM1_ESM.pdf]

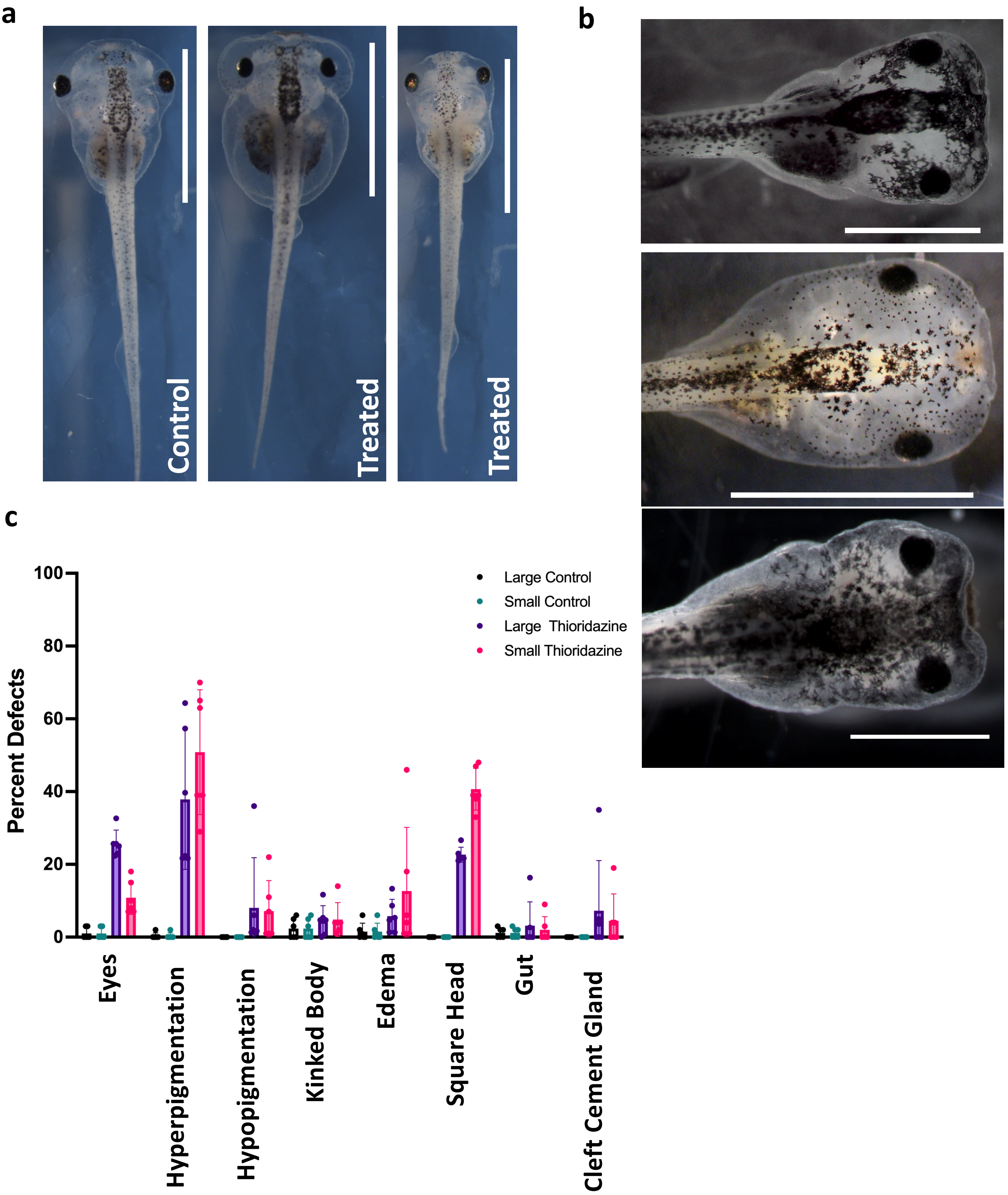

Supplemental Figure 1. Additional images of thioridazine treated tadpoles and additional noted defects. (A) Whole body images of thioridazine treated animals. (B) Variations in square head phenotypes. (C) Quantification of all observed defects from thioridazine treatment in large and small groups with their respective controls.

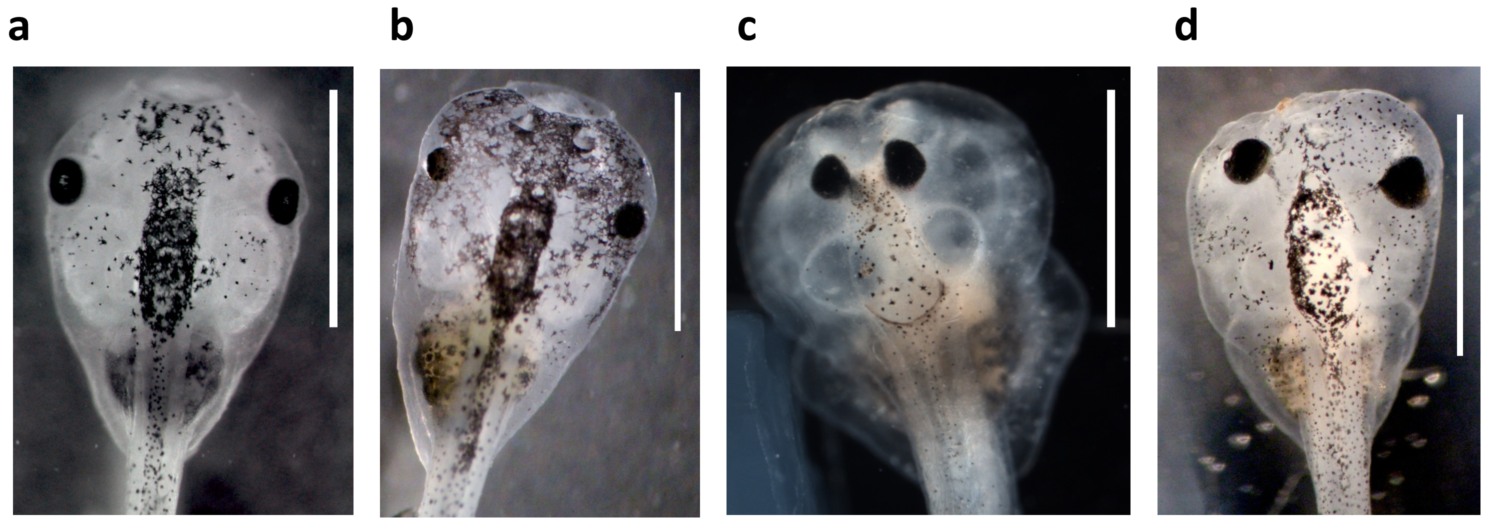

Supplemental Figure 2. Supplementary images of tadpoles treated with different teratogens. (A) Example control embryo. Example of forskolin (B), nicotine (C), and kir6.1 (D) treated embryos.

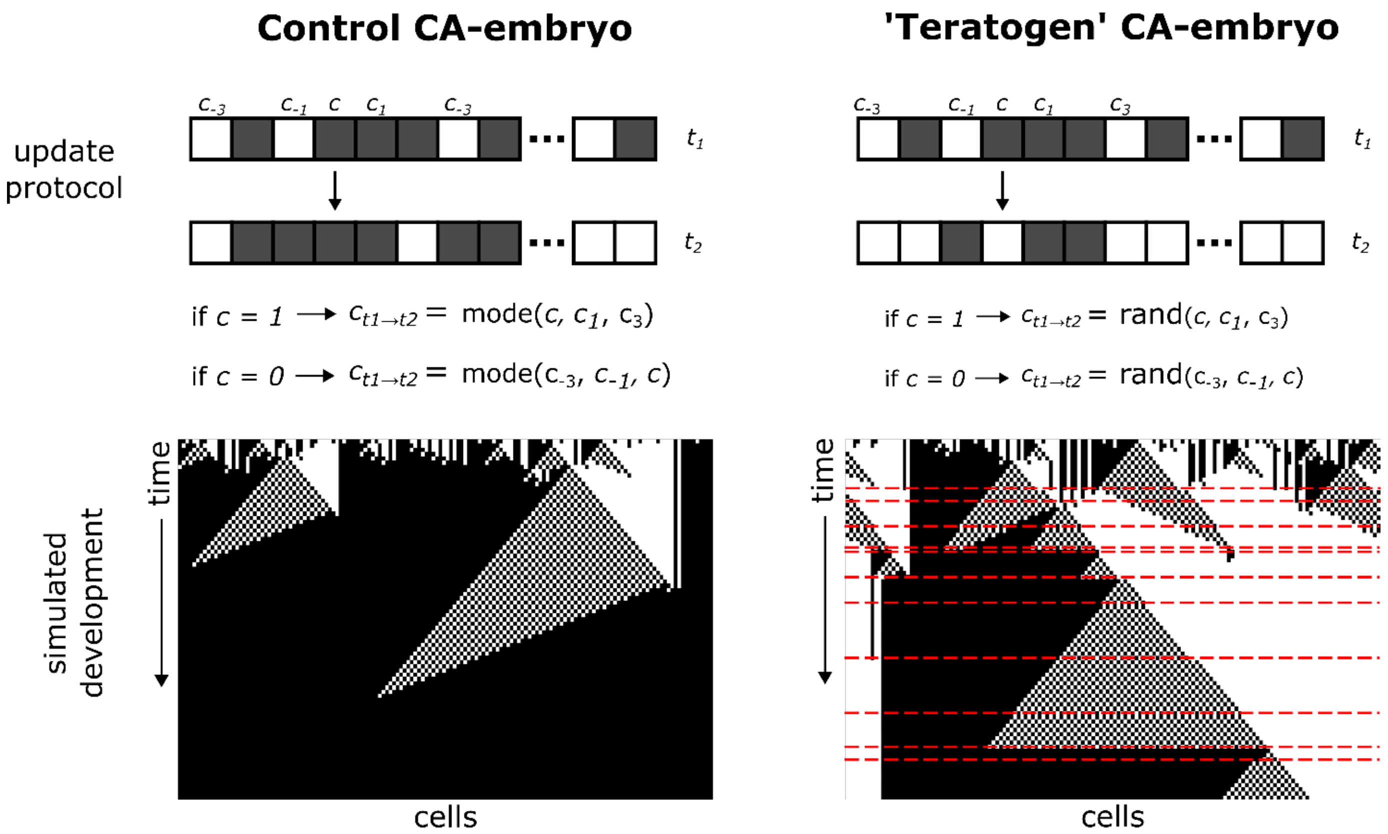

<u>Supplemental Figure 3.</u> A visual example of how the control and 'teratogen-exposed' ECAs update from one time step to another. (Left) Normally, cells follow the GKL rule [84], successfully solving the majority problem. There is an initial distribution of ones (black) and zeros (white) with more ones than zeros. The ECA evolves (top to bottom) and converges to all ones. (Right) In the presence of noise (red dashed line), the rules shift (and stay shifted) to random updating, and the ECA fails to solve the majority problem. There is the same majority in the right as in the left, but at the end of the simulation not all cells are one/black. These examples were generated as singletons, and no collective effects are shown here.

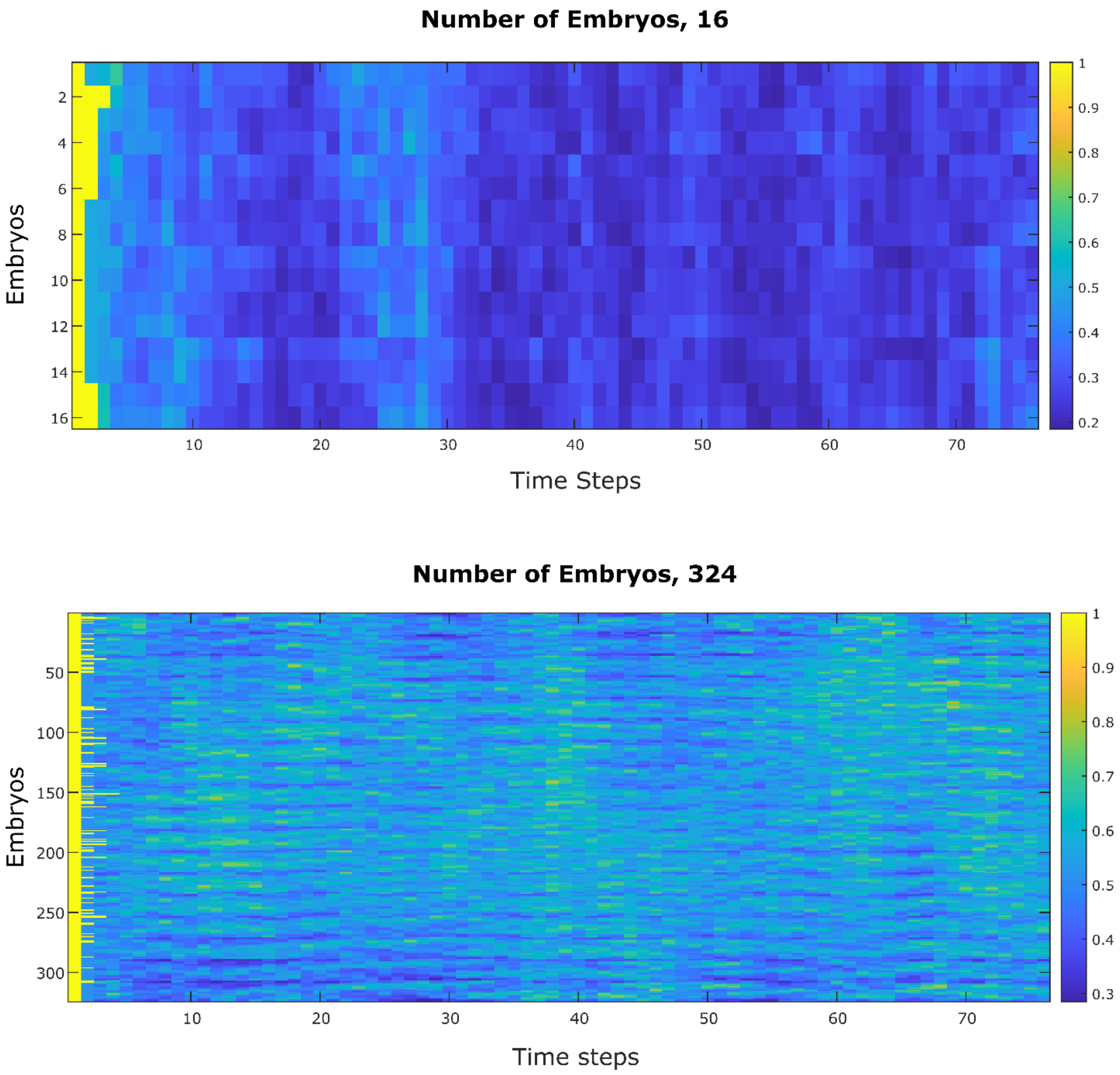

Supplemental Figure 4. Two representative examples of health values over time. In each graph, the y-axis is embryos in the group and the x-axis is time. At each time point the health value of the embryo is reported as a color (yellow is more health and blue is more unhealthy). For low numbers of total embryos, health values quickly collapse to low values. This can be seen in the top graph as the colors slowly drift to a dark blue color. Conversely, under the same conditions, large numbers of embryos better resist noise, resulting in healthy, although not perfectly healthy, embryos. This can be visually seen in the lower graph, where the health values are more yellow than the upper graph, meaning higher health. While not a central area of investigation for this study, it is particularly interesting how health and noise balance one another.

a **local vs CEMA survival rates**

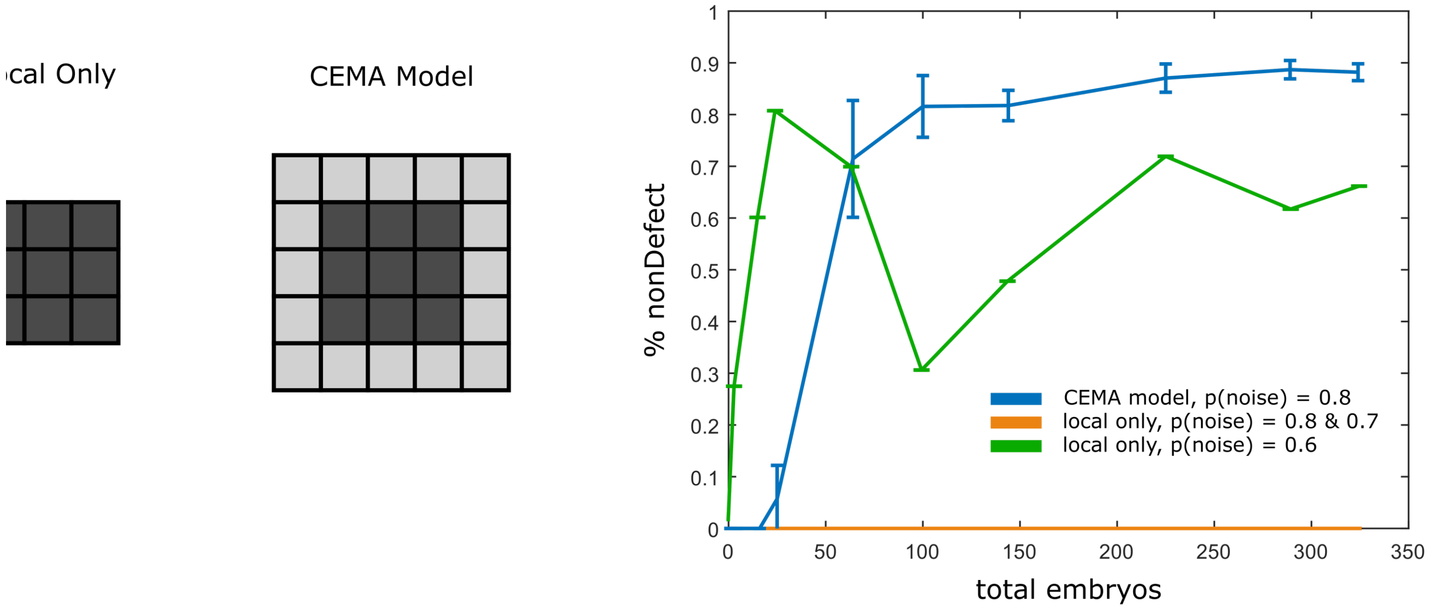

b **health threshold evaluation**

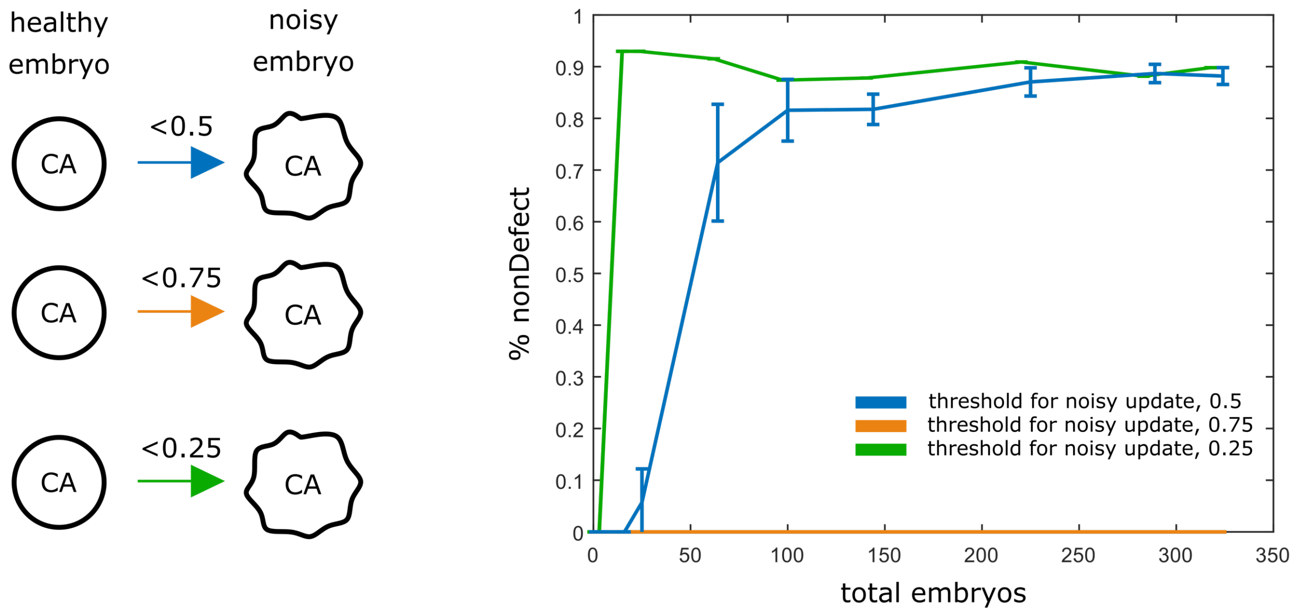

Supplemental Figure 5. (A) Left, a schematic of two tested conditions – first with only neighbor's interactions involved in the simulations and second with neighbor's neighbors' involved. Right, a graph showing how these two conditions (orange and blue, respectively) effect how the cohort resists noise. Even when lowering noise (green), small, local-only interactions are not enough to resist noise. (B) Left, a schematic showing how health threshold values were tested. Right, a graph showing how these three conditions altered the simulations while keeping all other parameters set.

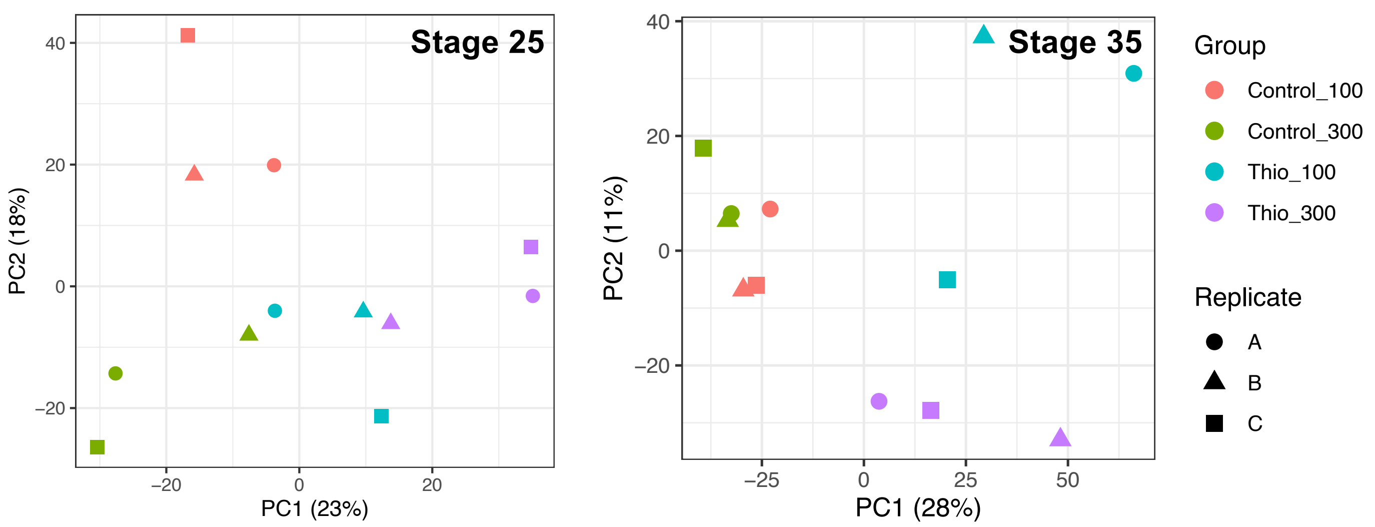

<u>Supplemental Figure 6.</u> Principal component analysis of RNA-sequencing data for Stage 25 and Stage 35 *Xenopus* housed in small (100-embryo) and large (300-embryo) group sizes in control or thioridazine-treated media.

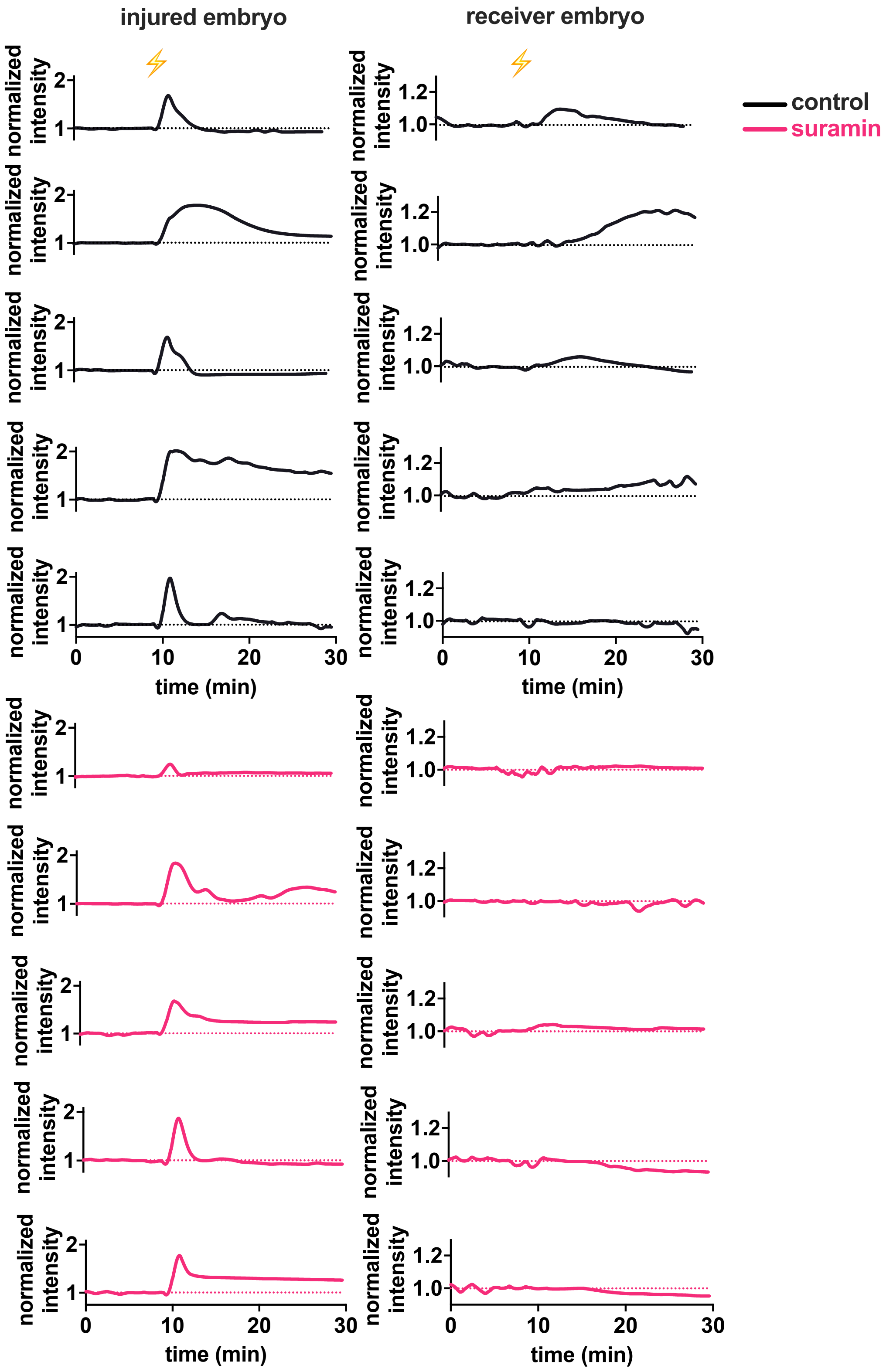

Supplemental Figure 7. Intensity vs. time plots for control and suramin-treated embryos. Baseline (0-10 minutes) and post-injury (10-30 mins) traces are shown for the injured and receiver embryos. The time point of injury is denoted by the lightning bolt.

a

Control_300 vs. Control_100	Thio_300 vs. Thio_100
LOC108699620	crisp1.5.S
LOC108706197	eif5b.L
LOC121398066	astl2f.S
ambp.L	LOC108699319
LOC108717648	xa-1.L
LOC121399129	slc7a5.L
LOC108696295	hnf4a.S
apoc1.L	astl3b.1.L
selenop1.L	slc26a4.3.S
LOC108696199	LOC121398849
fga.S	xepsin.S
LOC108697756	h1-0.L
rpl37.S	LOC108709516
LOC121393615	rpl35a.S
hba3.L	ddit3.L
atp5mg.L	atp6ap1.1.L
LOC121397762	astl2d.2.L
rpl38.L	h1-0.S
dbi.S	LOC108701262
fam118b.S	
dan4l.L	
apoc2.L	
rpl38.S	
rps8.S	
fgb.S	
LOC121395005	
rps28p9.L	
hrg.L	
edf1.L	
LOC108704296	
LOC108697889	
kng1.S	

UP (FDR<0.05)
DOWN (FDR<0.05)

b

Gene	Product
crisp1.5.S	cysteine-rich secretory protein 1 gene 5 S homeolog
eif5b.L	eukaryotic translation initiation factor 5B L homeolog, transcript variant X2
astl2f.S	astacin-like metallo-endopeptidase 2 gene f S homeolog, transcript variant X2
xa-1.L	anterior and ectodermic-specific protein L homeolog
slc7a5.L	solute carrier family 7 member 5 L homeolog
astl3b.1.L	astacin-like metallo-endopeptidase 3 gene b1 L homeolog
slc26a4.3.S	solute carrier family 26 member 4 gene 3 S homeolog
xepsin.S	epidermis specific serine protease S homeolog
h1-0.L	H1.0 linker histone L homeolog
rpl35a.S	ribosomal protein L35a S homeolog
ddit3.L	DNA damage inducible transcript 3 L homeolog, transcript variant X1
atp6ap1.1.L	ATPase, H+ transporting, lysosomal accessory protein 1, gene 1 L homeolog, transcript variant X1
astl2d.2.L	astacin-like metallo-endopeptidase 2 gene d2 L homeolog
h1-0.S	H1.0 linker histone S homeolog
LOC108699319	embryonic protein UVS.2-like
LOC121398849	ATP-dependent RNA helicase DHX8-like
LOC108701262	insulin-like growth factor-binding protein 2
LOC108709516	uromodulin
hnf4a.S	hepatocyte nuclear factor 4 alpha S homeolog, transcript variant X1

Supplementary Table 1. Differentially expressed genes (FDR < 0.05) for Stage 35 *Xenopus* housed in small (100-embryo) and large (300-embryo) group sizes in control or thioridazine-treated media. (A) All genes differentially expressed in each of the group sizes. (B) List of significantly differentially expressed genes from thioridazine treated large vs small groups with their known products.
