# Supplementary material for: Embryos assist morphogenesis of others through calcium and ATP signaling mechanisms in collective teratogen resistance
Source: Nat Commun. 2024 Jan 17;15:535. doi: 10.1038/s41467-023-44522-2 (PMC10794468; doi:10.1038/s41467-023-44522-2)
Supplement: Supplementary file 3 — Description of Additional Supplementary Files [file 41467_2023_44522_MOESM3_ESM.pdf]

Description of Additional Supplementary Files

File Name: Supplementary Movie 1
Description: Calcium waves travels across an injured embryo. (top) and across adjacent, un-injured embryos (middle and bottom). Bright spot on injured embryo is the site of injury. Injured embryo is on left and un-injured embryos is on right (middle). Injured embryo is in the center and surrounded by un-injured embryos (bottom).

File Name: Supplementary Movie 2
Description: In a pair of embryos, a calcium wave travels from an injured embryo to an adjacent, uninjured embryo.

File Name: Supplementary Movie 3
Description: In a group of 10 embryos, multiple calcium waves travel from an injured embryo to adjacent, uninjured embryos.

File Name: Supplementary Movie 4
Description: ATP is sufficient to elicit a calcium response. The addition of ATP into a channel with a single embryo is able to initiate a calcium response in the un-injured embryo. Site of ATP addition is labeled on the left side of the channel while an un-injured embryo was placed on the right side.
